# Impaired Mitochondrial Morphology and Functionality in *Lonp1*^wt/−^ Mice

**DOI:** 10.3390/jcm9061783

**Published:** 2020-06-08

**Authors:** Anna De Gaetano, Lara Gibellini, Elena Bianchini, Rebecca Borella, Sara De Biasi, Milena Nasi, Federica Boraldi, Andrea Cossarizza, Marcello Pinti

**Affiliations:** 1 Department of Life Sciences, University of Modena and Reggio Emilia, 41125 Modena, Italy; anna.degaetano@unimore.it (A.D.G.); borafe24@unimore.it (F.B.); 2 Department of Medical and Surgical Sciences of Children and Adults, University of Modena and Reggio Emilia, 41125 Modena, Italy; lara.gibellini@unimore.it (L.G.); rebeccaborella7993@gmail.com (R.B.); debiasisara@yahoo.it (S.D.B.); andrea.cossarizza@unimore.it (A.C.); 3 Department of Surgery, Medicine, Dentistry and Morphological Sciences, University of Modena and Reggio Emilia, 41125 Modena, Italy; elena.bianchini.88@gmail.com (E.B.); milena.nasi@unimore.it (M.N.)

**Keywords:** Lon protease, CODAS, mitochondria, mouse model, enterocytes, mouse embryonic fibroblasts

## Abstract

LONP1 is a nuclear-encoded mitochondrial protease crucial for organelle homeostasis; mutations of *LONP1* have been associated with Cerebral, Ocular, Dental, Auricular, and Skeletal anomalies (CODAS) syndrome. To clarify the role of LONP1 in vivo, we generated a mouse model in which *Lonp1* was ablated. The homozygous *Lonp^−/−^* mouse was not vital, while the heterozygous *Lonp1^wt/−^* showed similar growth rate, weight, length, life-span and histologic features as wild type. Conversely, ultrastructural analysis of heterozygous enterocytes evidenced profound morphological alterations of mitochondria, which appeared increased in number, swollen and larger, with a lower complexity. Embryonic fibroblasts (MEFs) from *Lonp1^wt/−^* mice showed a reduced expression of *Lonp1* and *Tfam*, whose expression is regulated by LONP1. Mitochondrial DNA was also reduced, and mitochondria were swollen and larger, albeit at a lesser extent than enterocytes, with a perinuclear distribution. From the functional point of view, mitochondria from heterozygous MEF showed a lower oxygen consumption rate in basal conditions, either in the presence of glucose or galactose, and a reduced expression of mitochondrial complexes than wild type. In conclusion, the presence of one functional copy of the *Lonp1* gene leads to impairment of mitochondrial ultrastructure and functions in vivo.

## 1. Introduction

LONP1 protease is a nuclear-encoded protein located in the mitochondrial matrix, belonging to the ATPase associated with diverse cellular activities (AAA+) superfamily [1]. LONP1 is highly conserved throughout evolution from *archea* to mammals and it is ubiquitously expressed in different cells and tissues [2,3,4].

LONP1 is composed of three domains: the N terminus interacts with the substrates, the AAA+ domain binds and hydrolyzes ATP and the P terminus contains the proteolytic active site [5,6,7]. The main function of LONP1 is to degrade misfolded, oxidized and damaged proteins but it also actively participates in other mitochondrial functions, modulating the amount of protein involved in mitochondrial structure and mtDNA maintenance such as the Mitochondrial transcription factor A (TFAM) [8,9,10,11]. Protein degradation mediated by LONP1 can modulate the respiratory process [12,13] and the energetic metabolism [8,14]. LONP1 is a stress response protein, as its level increases in the presence of hypoxia, reactive oxygen species (ROS) and reticulum endoplasmic (ER)-stress, and its down-regulation impairs response to stress and alters mitochondrial morphology [14,15,16,17,18,19,20]. Finally, LONP1 displays chaperon activity and is required for maturation of several mitochondrial proteins [12,21].

Modified amounts and activity of LONP1 are associated with several diseases such as mitochondrial encephalomyopathy, lactic acidosis, and stroke-like episodes (MELAS), myoclonic epilepsy with ragged-red fibers (MERRFs), hereditary spastic paraplegia, Friedrich ataxia, hereditary paraganglioma and Highly Active Antiretroviral Therapy (HAART) related lipodystrophy [22,23,24,25,26,27]. LONP1 is upregulated in different types of cancer, and its upregulation makes cancer cells resistant to new environmental conditions, such as hypoxia, typical of malignant tumors [8,28,29,30,31,32,33].

Heterozygous and compound heterozygous mutations in AAA+ and proteolytic domains of the *LONP1* gene are linked to cerebral, ocular, dental, auricular, and skeletal anomalies syndrome (CODAS; MIM 600373), a rare recessive disorder diagnosed in 19 cases since its first description in 1991 [34,35,36,37,38,39]. CODAS is characterized by several congenital anomalies including short stature, ossification of epiphyseal and developmental delay, coronal clefts, dislocated hips, and craniofacial defects (median nasal groove, ptosis, bilateral early onset cataracts, anomalous ears and hearing loss, abnormal cusp morphology and delayed tooth eruption). The disease has a severe impact on the life of the affected patients, leading in some cases to premature death [39]. Skeletal abnormalities and cataracts are pathognomonic of CODAS, whereas other anomalies are in common with mitochondrial diseases [39,40]. Other biallelic variants in *LONP1* gene do not cause CODAS, but determine profound neurodegeneration with progressive cerebellar atrophy, associated with pyruvate dehydrogenase deficiency and metabolic disfunctions [41], or classical mitochondrial diseases, such as Leigh disease [40]. 

Deficiency of LONP1 has been studied in a mouse model, and it has been demonstrated that homozygous deletion of this gene is embryonically lethal, because of a progressive loss of mtDNA which finally leads to the death of fetus at 7.5 days post coitum. The heterozygous *Lonp1^wt/−^* mouse is viable and fertile, but this model has only partially been characterized [32], showing that haploinsufficiency of *Lonp1* abrogates ischemic preconditioning cardio-protection [42]; the analysis of the proteome of mouse embryonic fibroblasts (MEFs) suggests that the *Lonp1^wt/−^* genotype determines enhanced respiratory dysfunction [43].

We have previously shown that LONP1 downregulation determines a severe impairment of mitochondrial morphology and functionality in colon cancer cells, and that this impacts on epithelial mesenchymal transition in colon cancer. Thus, in this study we generated and characterized a heterozygous *Lonp1^wt^*^/-^ mouse model, focusing our attention of the effects of *Lonp1* haploinsufficiency on mitochondria morphology and function of colon enterocytes, and on the functional effects on mitochondria of MEFs.

## 2. Materials and Methods

### 2.1. Generation and Genotyping of Lonp1^wt/−^ Mice

The *Lonp1^wt/−^* mouse (background C57BL/6) was provided by Biogem Srl (Ariano Irpino, Italy) by generating a knock-out allele encompassing exons 5 and 8 (Figure S1). Genotyping was performed by polymerase chain reaction (PCR) on 20–50 ng of genomic DNA obtained from ear samples using the commercial Easy-DNA™ gDNA Purification Kit (Thermo Fisher Scientific, Waltham, MA, USA) amplified in 25 μL of reaction mixture having this composition: 1X GoTaq Flexy Buffer (Promega Corporation, Madison, WI, USA), 2 mM MgCl_2_, 0.2 mM of each dNTP (Promega Corporation), 0.03 U of GoTaq G2 Flexy DNA Polymerase and 0.2 μM of each primer. The cycling parameter protocol included an initial step of denaturation at 95 °C for 1 min followed by: 2 cycles at 95 °C 15 s, 64 °C 15 s, 72 °C 1 min; 2 cycles at 95 °C 15 s, 61 °C 15 s, 72 °C 1 min; 20 cycles at 95 °C 15 s, 58 °C 15 s, 72 °C 1 min; 10 cycles at 95 °C 15 s, 55 °C 15 s, 72 °C 1 min and a final extension step at 72 °C for 10 min. The following primers were used: forward 5′-cagggaagaaactgaagtcaggc-3′, reverse 5′-cactctggttcatggccacc-3′, producing a 925 bp product only for knock-out allele, detected by agarose gel electrophoresis. 

### 2.2. Ethics Statement

All animal procedures were approved in accordance with Italian law protecting animals used for scientific purposes (authorization n°253/2017-PR, released on 21st March 2017 by the Italian Ministry of Health). 

### 2.3. Isolation of Organs and Tissue 

Mice were subjected to euthanasia and sacrifice by cervical dislocation, then placed on their back on a thick pad of blotting paper. After soaking fur in 70% ethanol, skin was cut with scissors and the organs of interest were explanted and analyzed for any morphological variation, then weighed and measured for dimensions. Heads and red organs were harvested from embryos during the isolation of MEFs. Finally, all the tissues were stored in liquid nitrogen or treated for subsequent applications. 

### 2.4. Isolation and Culture of MEFs

Mouse embryos at 12.5 to 15.5 days post coitum were harvested from a pregnant female and dissected into sterile phosphate buffered saline (PBS) added with Penicillin/Streptomycin (10,000 U/mL; Thermo Fisher Scientific). After the removal of the visible internal organs and of the head, the carcasses were minced using a scalpel and digested with 0.25% trypsin-EDTA (Thermo Fisher Scientific) added with Penicillin/Streptomycin at 37 °C for 20 min. The obtained cell suspensions were transferred into 25 cm^2^ flasks and cultured in Dulbecco’s Modified Eagle Medium (DMEM) Glutamax (Thermo Fisher Scientific) supplemented with 10% fetal bovine serum (FBS; Thermo Fisher Scientific) and Penicillin/Streptomycin. After the confluence, MEFs were grown in 75 cm^2^ flasks and used in experiments at 5th passage. The galactose medium used in bioenergetics experiments is composed of DMEM without glucose (Thermo Fisher Scientific) supplemented with 10% FBS, 5 mM galactose, 5 mM pyruvate and Penicillin/Streptomycin. Cells have been grown in galactose medium for 9 days. 

### 2.5. Quantification of Mitochondrial DNA

Total DNA was isolated from 25 mg of fresh or stored tissue by using QIAamp^®^ DNA Mini Kit (QIAGEN, Hilden, Germany) following manufacturer’s instructions. Amount of DNA was measured using the NanoDrop ND-1000 (Thermo Fisher Scientific) and samples were diluted to 50 ng/μL.

The amount of mtDNA was quantified by real-time in tissues and by droplet digital PCR (ddPCR) in MEFs. Real time PCR was carried out on CFX96 Touch™ Real-Time PCR Detection System (Bio-Rad Laboratories, Hercules, CA, USA) and the reaction mixture had this composition in 10 μL of final volume: 1 μL of DNA samples, 1X SsoAdvanced universal SYBR^®^ Green super-mix (Bio-Rad Laboratories) and 15 pM of each primer. The thermal protocol includes an initial step of polymerase activation and DNA denaturation at 95 °C for 2 min, followed by 40 cycles of denaturation at 95 °C 5 s, annealing/extension and plate read at 60 °C 20 s; after, a melting curve with 0.5 °C increment 5 s/step between 65 °C and 95 °C was determinate. mtDNA amount was normalized to Tubulin, as relative quantification. The following primers were used: *Tubulin* forward: 5′-GCCAGAGTGGTGCAGGAATA-3′, Tubulin reverse: 5′-TCACCACGTCCAGGACAGAGT-3′, mtDNA forward: 5′-CATTTGCAGACGCCATAA-3′, mtDNA reverse; 5′-TGGGTGTGGTATTGGTAGGG-3′. For every point and every group of the investigation, tissues belonging to three mice were evaluated and cycle threshold (Ct) values were used to calculate the ΔCt between mtDNA and the control gene and used to calculate the mtDNA amount. 

The reaction mixture of ddPCR in 20 μL of final volume is composed of: 20 ng of DNA samples, 2X ddPCR Supermix for Probes (Bio-Rad Laboratories), 1 μL of PrimePCR™ ddPCR™ Expression Probe Assay mt-Nd1 (FAM fluorescence), 1 μL of PrimePCR™ ddPCR™ Expression Probe Assay Tubb2b (HEX fluorescence). Thermal cycling conditions are: 95 °C for 10 min, followed by 38 cycles of 94 °C for 30 s and 55 °C for 1 min, then 98 °C for 10 min. Droplet reading is performed on QX200 ddPCR droplet reader (Bio-Rad laboratories). Analysis are performed using QuantaSoft Analysis software (version 1.7.4.0917, Bio-Rad laboratories).

### 2.6. Immunoblot

Total cellular and tissue proteins were obtained by lysing cells and homogenizing tissues with RIPA buffer (50 mM Tris, pH 7.5, 0.1% Nonidet P-40, 0.1% deoxycholate, 150 mM NaCl, 4 mM EDTA, and protease inhibitors cocktail). The tissues were maintained in ice and homogenized for ~5 min until they were fully dissociated, while cellular pellets were dissociated using syringe and needles. Samples were kept in ice for 30 min, and then they were centrifuged at 15,000× *g* for 30 min at 4 °C. Proteins were quantified by Bradford assay (Bio-Rad Laboratories). Samples (30–50 µg) were separated in 4–12% Bolt Bis-Tris precast gels (Thermo Fisher Scientific) and transferred onto nitrocellulose or polyvinylidene fluoride (PVDF, for OXPHOS complexes) membranes (Bio-Rad Laboratories) by using methanol transfer buffer. After blocking with 5% Blotting-Grade Blocker (Bio-Rad Laboratories), the membranes were incubated with the following primary antibodies: custom anti-LonP1 (1:500) from Primm (Milan, Italy), anti-TFAM (1:2000; Abnova Corporation, Taipei, Taiwan), anti-OXPHOS complexes (1:250; Abcam, Cambridge, UK) anti-α-Tubulin (1:1000; Sigma-Aldrich, Saint Louis, MO, USA), anti-ACO2 (1:1000; Abnova Corporation), anti-GRP75 (1:500; Santa-Cruz Technologies, Dallas, TX, USA), anti-SOD2 (1:500; Abcam), anti-TOM20 (1:500; Santa-Cruz Technologies) and then washed and incubated with the secondary antibodies Goat Anti-Mouse IgG (H + L)-HRP Conjugate and Goat Anti-Rabbit IgG (H + L)-HRP Conjugate (1:5000; Bio-Rad Laboratories). Peroxidase activity was detected using Clarity™ Western ECL Blotting Substrates (Bio-Rad Laboratories). Images were acquired by using ChemiDoc MP (Bio-Rad Laboratories). The densitometric analysis was performed with Image Lab (version 6.1, Bio-Rad Laboratories). Bands were automatically detected using the “Lane and Bands” tool, and the band intensities analyzed with Prism 8.0 (GraphPad Software, San Diego, CA, USA).

### 2.7. Hematoxylin and Eosin Staining

After explant, the tissues were washed with PBS, fixed with 4% paraformaldehyde in PBS for ~2 h and embedded in paraffin. Serial cross-sections were cut and then mounted onto slides. Hematoxylin and eosin (H&E) staining was performed on sections of 5 μm in order to analyze the morphological details of the tissues. 

### 2.8. Confocal and Stimulated Emission Depletion (STED) Microscopy

MEF samples were seeded at 3 × 10^5^ cells/well in 6-well plates on glass coverslips, and fixed with 4% paraformaldehyde for 10 min. After fixation, cells were permeabilized with 0.1% Triton X-100 in PBS for 5 min and blocked with 3% bovine serum albumin (BSA) in PBS for 30 min, at room temperature. Incubation with primary antibodies were performed for 1 h at room temperature. After washing with 3% BSA in PBS, samples were incubated for 1 h at room temperature with secondary antibodies. Samples were then washed in PBS and stained with 0.5 μg/mL 4,6-diamidino-2-phenylindole (DAPI) (Sigma-Aldrich) in PBS for 5 min, washed again in PBS and mounted. Samples prepared for STED microscopy were not incubated with DAPI. Cells were observed and images were acquired at Leica TCS SP8 confocal laser scanning microscope (Leica Microsystems, Wetzlar, Germany). STED images were obtained by using a Leica TCS SP8 microscope (Leica Microsystems) equipped with a HC PL APO 100x/1.40 OIL, a white light laser (470–650 nm), STED 3X, FLIM, a 775 nm pulsed depletion laser and hybrid detectors. The following antibodies were used: anti-LonP1 (1:100; Primm), anti-TOM20 (1:50; Santa-Cruz Technologies), anti-dsDNA (1:100; Santa Cruz Technologies), goat anti-rabbit Alexa Fluor 488 (1:200; Thermo Fisher Scientific), goat anti-mouse Alexa Fluor 488 (1:200; Thermo Fisher Scientific), goat anti-mouse Alexa Fluor 546 (1:200; Thermo Fisher Scientific), Goat anti-Rabbit IgG (H + L) Alexa Fluor 647, Goat anti-mouse Alexa Fluor 594 (1:500; Thermo Fisher Scientific), goat anti-rabbit ATTO 647N (1:500; Abcam).

### 2.9. Electron Microscopy

Colon and cell pellet were processed as described previously with some modifications [44]. Briefly, samples were chemically fixed in a solution of 2.5% glutaraldehyde (Electron Microscopy Sciences) dissolved in 0.1 M cacodylate buffer at pH 7.4 for 12 h. Post fixation was performed for 1 h in 1% OsO_4_ using the same buffer. The specimens were dehydrated with ethanol in concentrations increasing to 100% and embedded in Epson 812 (Electron Microscopy Sciences, Hatfield, PA, USA). The resin polymerization was carried out at 60 °C for 2 days. Ultrathin sections (60–70 nm) were cut and mounted on 150-mesh copper grids and stained with UranyLess (Electron Microscopy Sciences) followed by lead citrate. Sections were observed with a FEI NOVA NanoSEM 450.

### 2.10. Morphological Analysis of Mitochondria

Morphological measurement of mitochondria was obtained using ImageJ (version 1.52p, National Institutes of Health, USA). Distinct mitochondria were manually traced on electron microscopy images of colon of adult mice and MEFs and the following parameters were analyzed: mitochondrial size; perimeter; aspect ratio (AR, length-to-width ratio) as [(major axis)/(minor axis)]; form factor (FF, reflecting the complexity aspect of mitochondria) as [(perimeter^2^)/(4π·surface area)]. 

### 2.11. Oxygen Consumption Rate (OCR) and Extracellular Acidification Rate (ECAR)

XFp Extracellular Flux Analyzer (Seahorse Biosciences—Agilent Technologies, Santa Clara, CA, USA) was used to assay OCR and ECAR. Cells were plated in number of 2 × 10^4^ cells/well 1 day before the experiment, and experiments were performed on a confluent monolayer. Measurements were performed under basal conditions and then after injections of oligomycin (1.0 µM), carbonyl cyanide 4-trifluoromethoxy-phenylhydrazone (FCCP, 1.0 µM), rotenone and antimycin A (0.5 µM of both). Data have been normalized to sample protein concentrations, following instructions provided by the manufacturer.

### 2.12. Cytofluorimetric Analyses

Flow cytometry was used to determine mitochondrial membrane potential (MMP), and mitochondrial superoxide production. For MMP, cells were incubated with tetramethyl-rhodamine, methyl ester (TMRM, 100 nM, Thermo Fisher Scientific) for 30 min at 37 °C, and valinomycin (10 µM, Sigma Aldrich) for 15 min at 37 °C was used to induce mitochondrial membrane depolarization. Mitochondrial superoxide production was assessed using MitoSox Red Mitochondrial Superoxide Indicator (mtSOX, 5 µM, Thermo Fisher Scientific) for 30 min at 37 °C and H_2_O_2_ (2 mM, Sigma Aldrich) for 30 min at 37 °C as positive control. Then, cells were analyzed by flow cytometry using an Attune NxT (Thermo Fisher Scientific). The net median fluorescence intensity (MFI) was analyzed subtracting the MFI of the unstained samples to the MFI of the stained samples to prevent the influence of autofluorescence. Finally, the net MFI value of a wild type (wt) sample was set to 100 and other samples were determined as relative MFI level according to the formula: (heterozygous (het) sample net MFI/wt sample net MFI) × 100. Data were analyzed with FlowJo software (version 9.9.6, Tree Star, Ashland, OR, USA).

### 2.13. Statistical Analysis

Statistical analyses were performed using Prism 8.0 (GraphPad Software). The results are expressed as the mean ± SEM. Non-parametric Mann-Whitney test was used to compare quantitative variables. *p* value < 0.05 was considered significant.

## 3. Results

### 3.1. Lonp1 Depletion Causes Early Embryonic Lethality

We previously observed that *LONP1* silencing causes cell death in vitro in colon carcinoma cells [8]. To obtain more insight on the role of *Lonp1* downregulation in vivo, we generated a mouse model in which *Lonp1* gene was ablated; we deleted the exons 5–8 of the gene, which encode the domains with ATPase and proteolytic activity. We first obtained a heterozygous mouse (*Lonp1^wt/−^*) for the targeted allele of *Lonp1*, which was viable and fertile. When intermated, we did not obtain any KO mouse (*Lonp1^−/−^*) pups at birth, confirming the previously observed early embryonic lethality of the depletion of *Lonp1* [32]. Thus, we focused our attention on the characterization of the heterozygous mouse.

### 3.2. Phenotypic Characterization of the Lonp1^wt/−^ Mouse

We first characterized the *Lonp1^wt/−^* mouse from the phenotypic point of view. We explored the distribution of animals and litters among different types of mating. The analysis included 51 litters, for a total of 229 animals, in particular: 27 litters (110 animals) from *Lonp1^wt/−^* × *Lonp1^wt^*^/*−*^ mating, 13 litters (67 animals) from *Lonp1^wt/−^* ♂ × *Lonp1^wt^*^/*wt*^ ♀ mating and 11 litters (52 animals) from *Lonp1^wt/wt^* × *Lonp1^wt^*^/*−*^ mating (Table 1). We analyzed the distribution of sex of the wt and heterozygous (het) animals in the litters, finding that animals were born at the expected Mendelian ratio, whatever the genotype of parents were (Table 2). The distribution of the number of pups born and the duration of pregnancy was similar for all the considered mating types (not shown).

**Figure 1:**
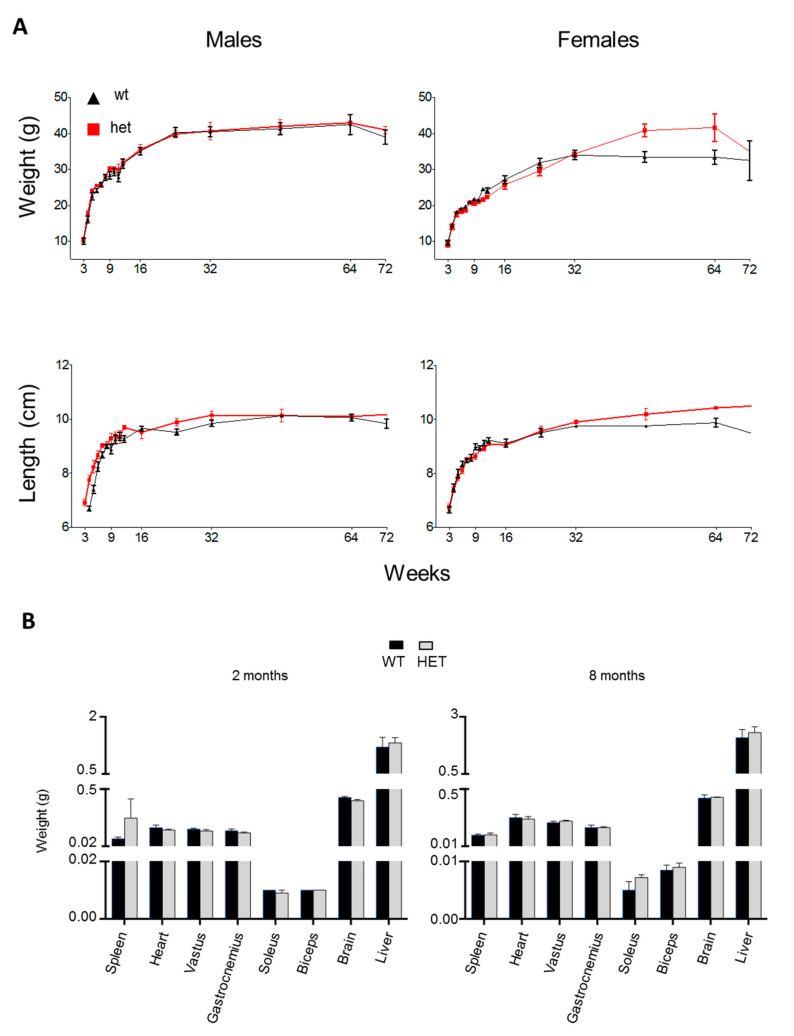
Phenotypic characterization of *Lonp1^wt/−^* mice. (**A**) Average weight and length of the body of heterozygous (het) (red line) and wild type (wt) (black line) mice. Measures started from the third week of life and were repeated every week until the 12th week, then at 4, 6, 8, 12, 16 and 18 months. Length was measured from nose to base of tail. Data are expressed as mean ± SEM of at least three different animals. (**B**) Analysis of weight of different organs and tissues. Measurements were taken after the organs explant in 2- and 8-months old mice. Data are expressed as mean ± SEM; n = 5 or more.

Weight and length of mice body were similar for wt and het mouse at any age considered (Figure 1A); no difference was noted when we compared only males or females. To gain more insight at organ level, we additionally analyzed the weight of different organs and tissues (spleen, heart, vastus, gastrocnemius, soleus, biceps, brain, liver) in young (2 months old) and adult (8 months old) animals. As shown in Figure 1B, we did not detect any genotype-dependent difference. 

Taken together, these data demonstrated that no gross phenotypic abnormalities could be observed in *Lonp1^wt/−^* mice.

### 3.3. LONP1 and mtDNA Levels in Wild Type and Lonp1^wt/−^ Mice

**Figure 2:**
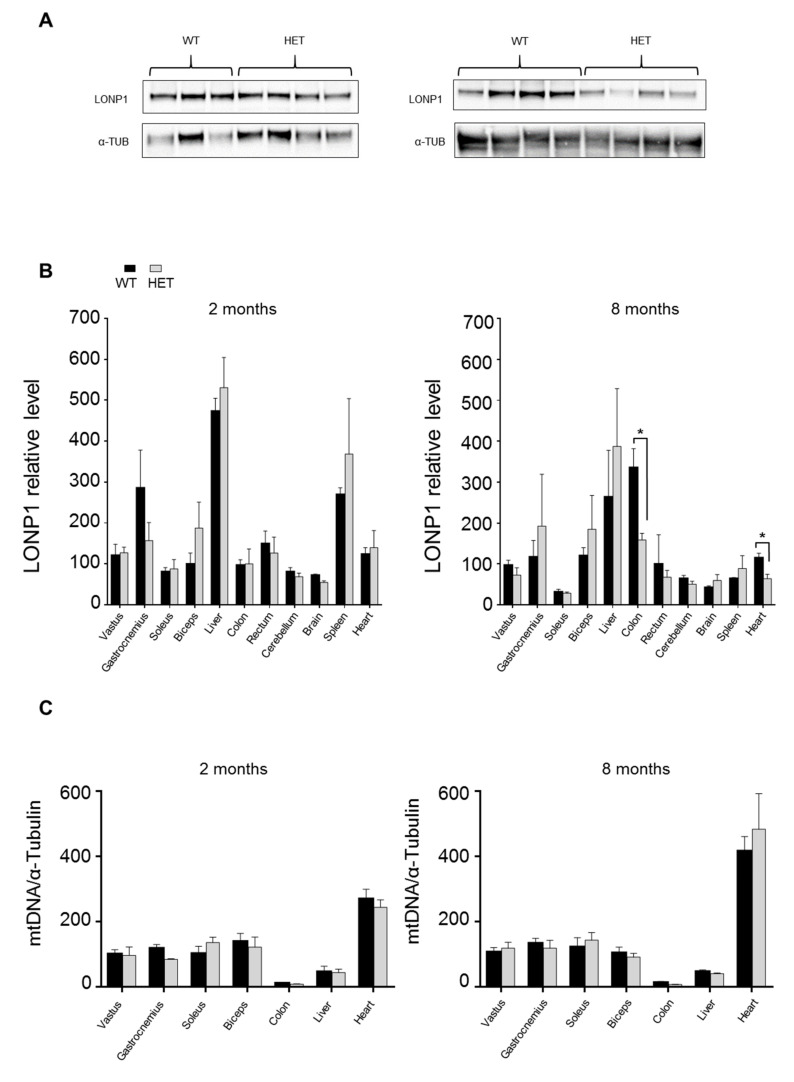
LONP1 protein expression and mtDNA amount in different tissues. (**A**) Representative examples of LONP1 expression in colon from wt and het mice. (**B**) Relative protein level of LONP1 in the indicated tissues from 2 and 8 months-old mice. Data are expressed as mean ± SEM; n = 7 or more. * = p < 0.05 (**C**) Quantification of mtDNA in different organs and tissues from 2 and 8 months-old mice; n = 3 or more. Data are expressed as mean ± SEM.

We next quantified expression of LONP1 protein in the same organs and tissues analyzed for the phenotypic characterization of het mouse; the analysis was performed in young (2 months old) and adult (8 months old) mice (Figure 2A,B and Figure S2). The expression of LONP1 is considerably variable among tissues, both in wt and *Lonp1^wt/−^* mice, with the highest levels observed in liver and spleen of young mice, and a lower but similar level observed in different muscles, colon, rectum, heart, cerebellum and brain. These levels were roughly maintained during aging, with the notable exception of the spleen, whose high levels in young mice tended to decline with age, and colon, whose level increases with age. Heterozygous mice expressed approximately the same amount of LONP1 protein as wt in most of the tissues and organs considered, with the exception of colon and heart of adult mice, in which LONP1 showed a significant reduction (53% and 45% respectively).

Since LONP1 binds to and is necessary for maintenance of mitochondrial DNA, we quantified the amount of mitochondrial DNA (mtDNA) in some of the tissues described above in young and adult mice (Figure 2C). The levels of mtDNA were similar in wt and het animals and changed considerably on the basis of the tissue taken into account, with the highest levels in heart and the lowest in the colon. We did not observe significant changes between young and adult mice. 

### 3.4. Histological Characterization of Lonp1^wt/−^ Mouse

**Figure 3:**
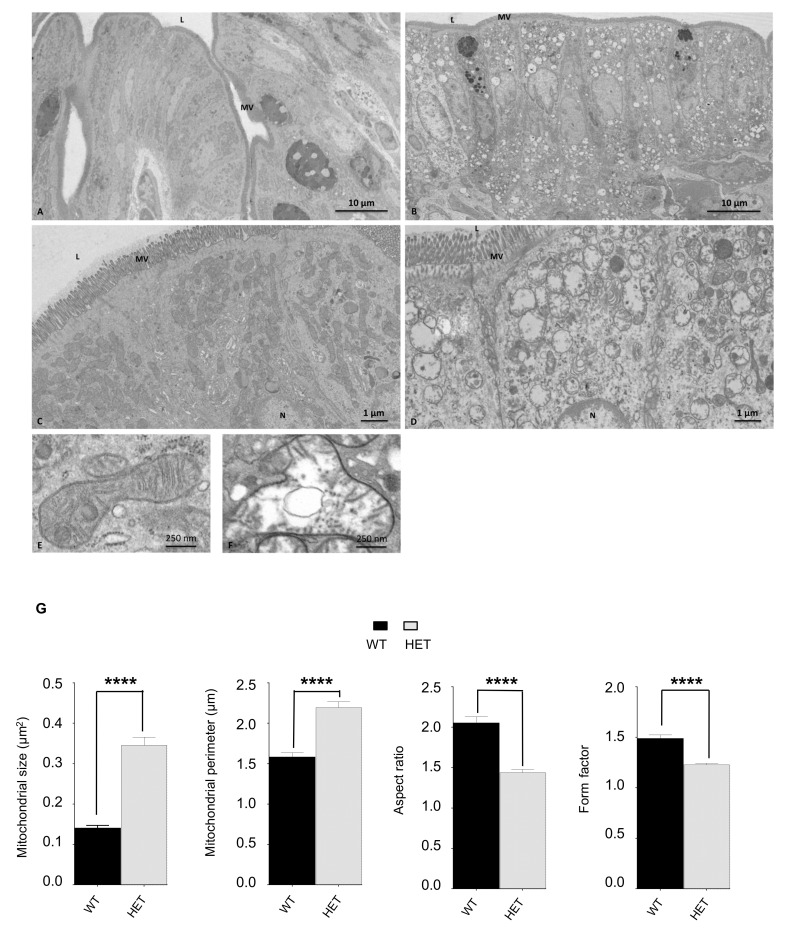
Ultrastructure analysis of mitochondria morphology of colon enterocytes of a wt and het mouse. (**A**–**F**) Ultrastructural features of mouse colon epithelium. **A**,**C**,**E**: wt; **B**,**D**,**F**: het. L = lumen; MV = microvilli; N = nucleus. (**G**) Analysis of mitochondrial size, perimeter, aspect ratio and form factor. Wt n = 203, het n = 153. Data are expressed as mean ± SEM. **** p < 0.0001.

Since LONP1 depletion has been associated with lower risk of developing colon cancer in mice, and since its downregulation deeply alters morphology and functionality of colon cancer cells in vitro, we decided to focus our attention on possible differences present in colon. We measured colon length, a sign of inflammation, but no changes have been observed (not shown). Then, we analyzed the colon from a histological point of view, but hematoxylin-eosin staining did not reveal gross differences between wt and het animals (Figure S3). Conversely, the ultrastructure analysis of enterocytes from the same samples showed an impairment of the structure of mitochondria, which appeared increased in number, swollen and larger in heterozygous mice, with a lower complexity and less organized cristae (Figure 3A–G). Quantitative analysis of mitochondrial area, a parameter associated with mitochondrial dimensions and perimeter, confirmed that mitochondria from *Lonp1^wt/−^* mice were larger, while lower values of aspect ratio and form factor correspond to objects that were, on average, longer and less circular (Figure 3G). 

### 3.5. LONP1 and TFAM Levels Are Lower in Het Embryos, and Lead to mtDNA Reduction and Mitochondrial Impairment

In order to get more insights into the functional effects of *Lonp1* haploinsufficiency, we analyzed the expression of *Lonp1* and the mitochondrial features of MEFs obtained from wt and *Lonp^wt/−^* mice. 

**Figure 4:**
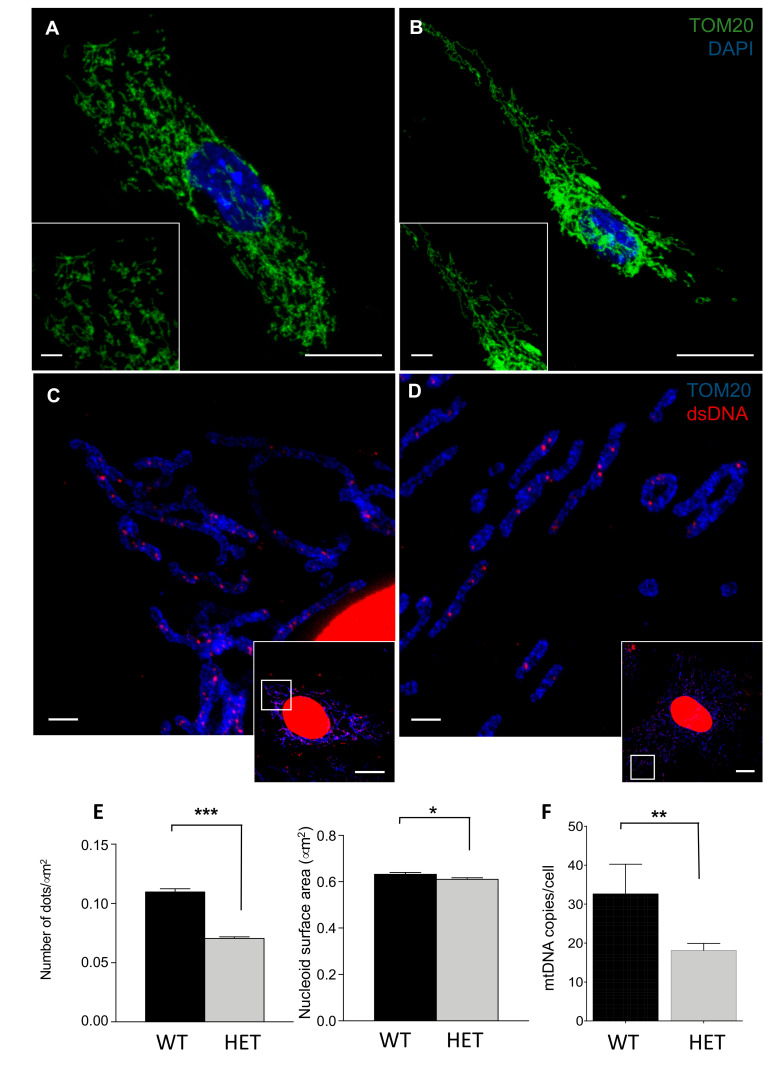
Imaging of mitochondrial and submitochondrial structures and mtDNA amount in embryonic fibroblasts (MEFs). (**A**,**B**) Confocal images of mitochondrial network from wt and het MEFs stained with anti-TOM20. **A**: wt; **B**: het. (Bars of larger panels are 1 µm; bars of smaller panels: 10 µm). (**C**,**D**) Stimulated emission depletion (STED0 images of mitochondrial DNA from wt and het MEFs stained with anti-TOM20 and anti-dsDNA. **C**: wt; **D**: het. (Bars of larger panels are 1 µm; bars of smaller panels: 10 µm). (**E**) Analysis of the number and the dimensions of nucleoids. (**F**) Mitochondrial DNA quantification of MEFs by droplet digital PCR. Data are expressed as mean ± SEM. * = p < 0.05, ** = p < 0.01, *** = p < 0.0001.

**Figure 5:**
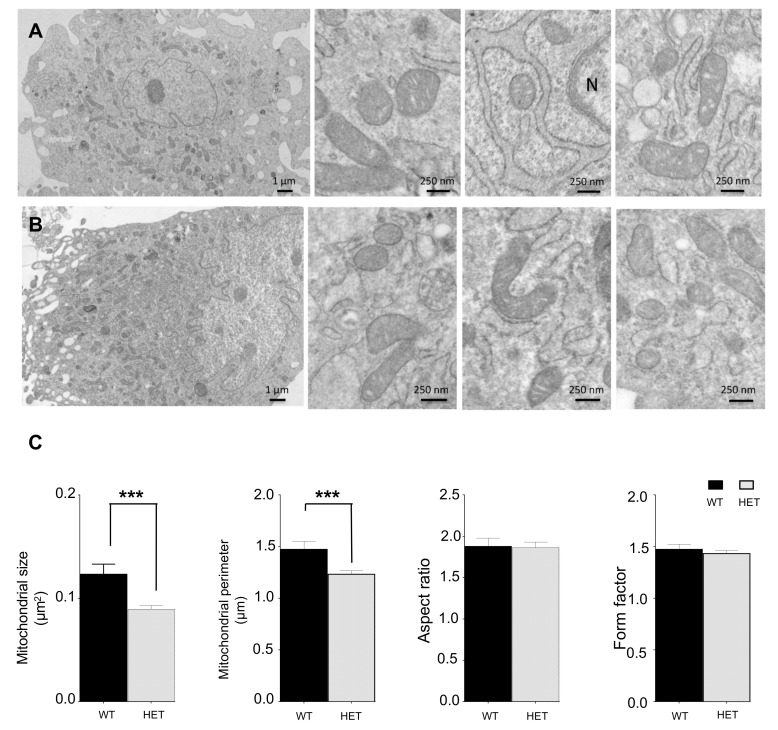
Ultrastructure analysis of mitochondria morphology of MEFs of wt and het mouse. Electron microscopy images of MEFs from wt (**A**) and het mouse (**B**). (**C**) Analysis of mitochondrial size, perimeter, aspect ratio and form factor. Wt n = 68, Het n = 192. Data are expressed as mean ± SEM. *** p < 0.001.

**Figure 6:**
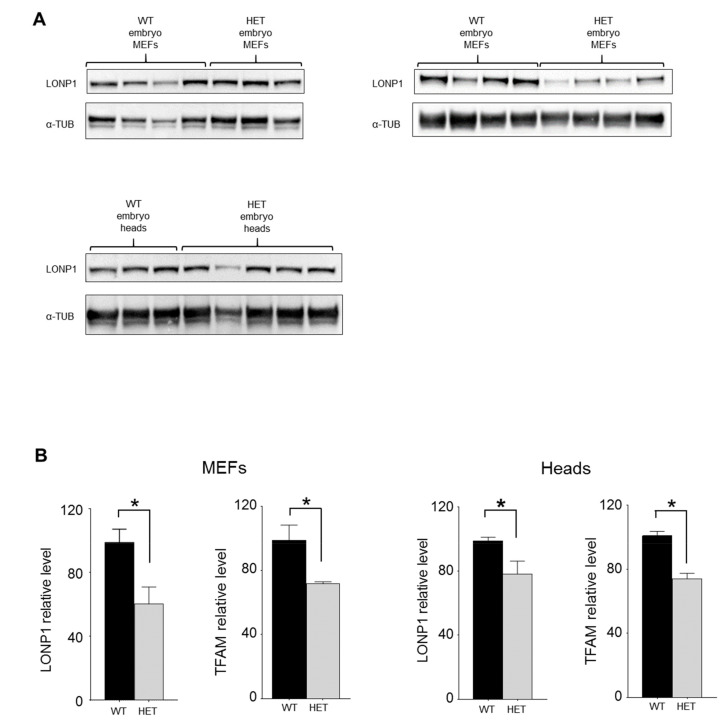
LONP1 and TFAM protein expression in heads of embryos and in MEFs. (**A**) Western blot analysis of LONP1 expression in heads of embryos and in MEFs. (**B**) Relative protein level of LONP1 and TFAM in heads of embryos and in MEFs. n = 3 or more. Data are expressed as mean ± SEM. * p < 0.05.

MEFs were cultured up to 55 days, and we did not observe any difference in the growth rate or number of replications between wt and het cells (Figure S4); cells showed a regular fibroblastic morphology. When we looked at mitochondrial morphology and ultrastructure of these cells, we observed that the organelles appeared mostly perinuclear in het MEFs, with isolated branches in the periphery of the cells (Figure 4A,B). Then, we analyzed nucleoids, whose LONP1 represents a component, within mitochondria through STED microscopy, and we observed a reduction in their number normalized to mitochondrial area, as well as in their dimensions, if compared to wt cells (Figure 4C–E). Mitochondrial DNA quantification by droplet digital PCR evidenced an average 44% reduction in mtDNA copies/cell in heterozygous MEFs (Figure 4F). The ultrastructure analysis revealed an increased number of mitochondria in heterozygous MEFs compared with wt, in a way similar to that observed in enterocytes from *Lonp^wt/−^* mice (Figure 5A,B). Quantitative analysis indicated a slight reduction in mitochondrial size and perimeter, without significant changes in aspect ratio and form factor (Figure 5C). We also observed an average 40% reduction of LONP1 protein expression in het MEFs; such reduction was also observed in heads from *Lonp^wt/−^* embryos, when compared to wt ones (Figure 6A,B). As LONP1 is involved in the regulation of cellular TFAM levels, we also evaluated the expression of this protein in the same samples. In a way similar to LONP1, TFAM showed a slight (30%), significant reduction in MEFs and embryo heads (Figure 6B and S5), in agreement with the reduction of mtDNA levels. 

**Figure 7:**
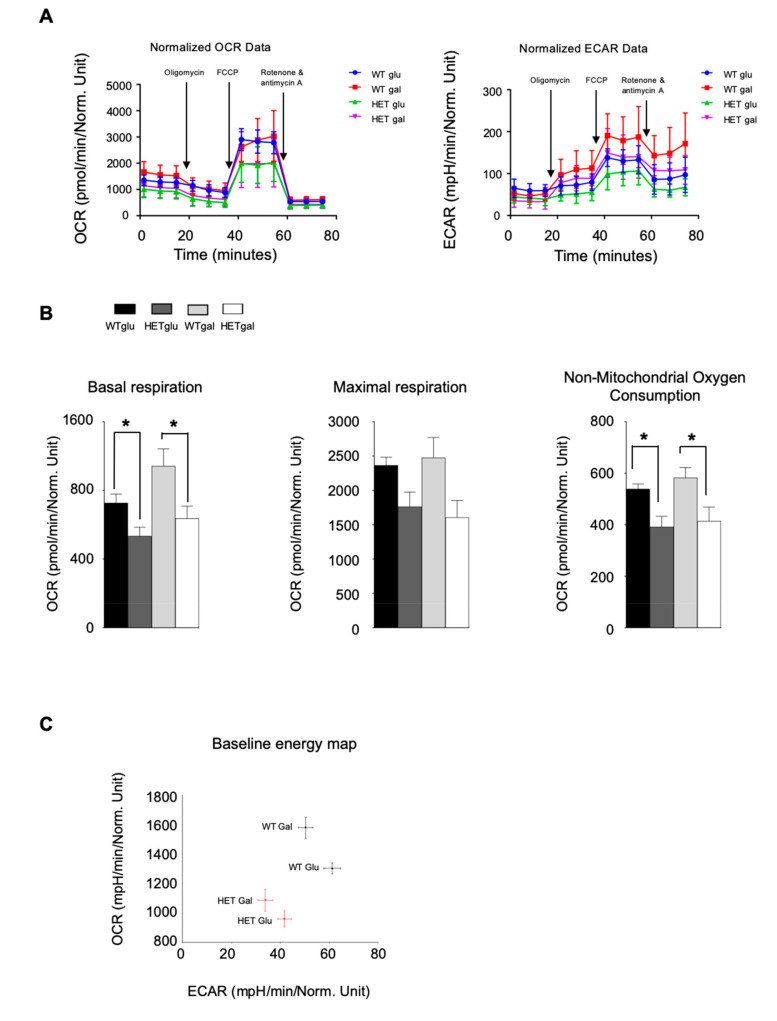

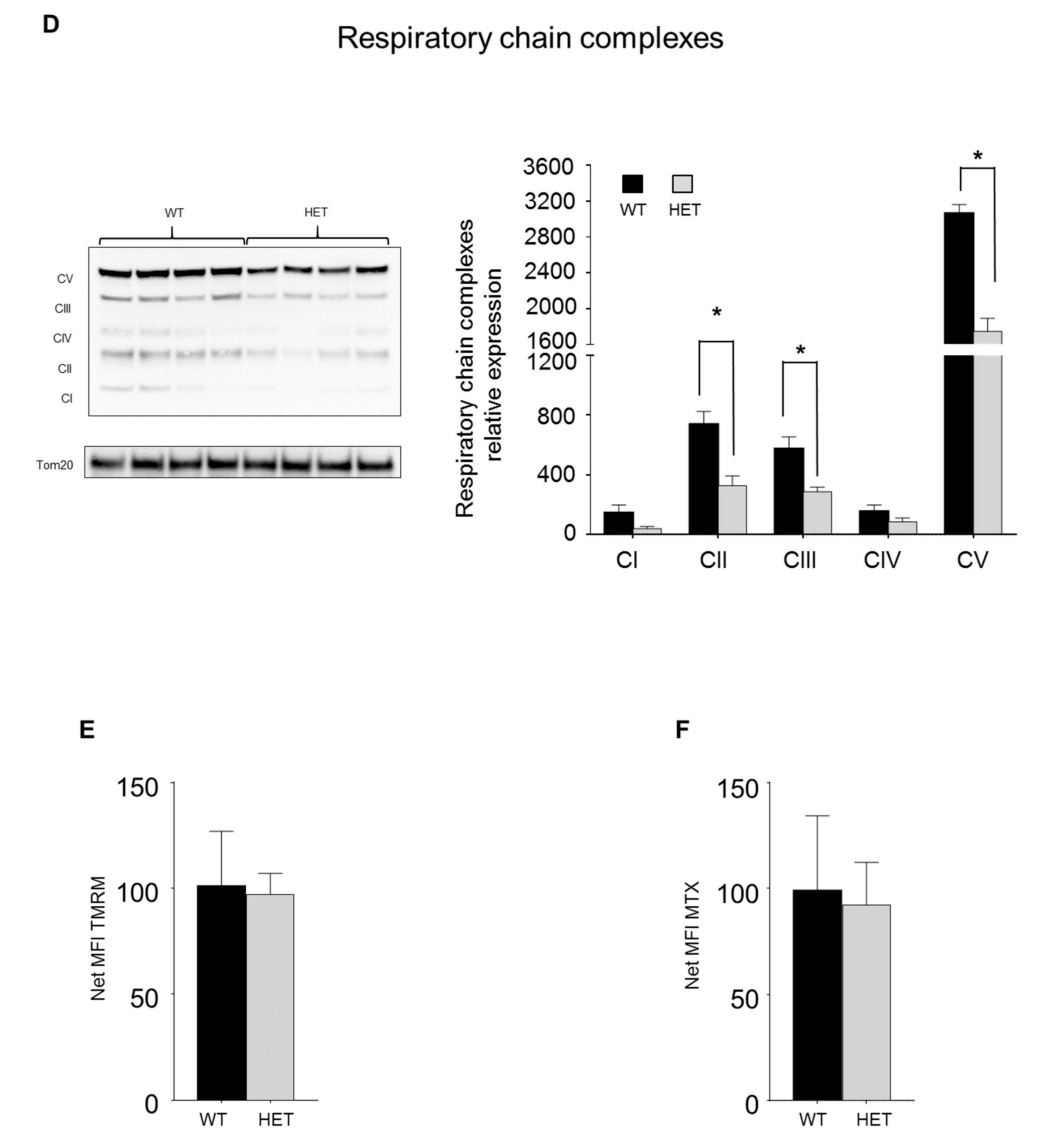
Analysis of mitochondrial functionality in wt and het cells. (**A**) Oxygen consumption rate (OCR) and extracellular acidification rate (ECAR) curves from the analysis of wt (blue and red) and het (green and purple) MEFs, obtained after the sequential addition of the indicated molecules. Data are expressed as mean ± SEM of three independent analyses. (**B**) Parameters of respiration in wt and het MEFs: basal respiration (left panel), maximal respiration, (central panel) and non-mitochondrial oxygen consumption (right panel). Data are expressed as mean ± SEM of three independent experiments. (**C**) Graph depicting OCR vs ECAR of wt and het cells grown in glucose or galactose. Data are expressed as mean ± SEM of three independent experiments. (**D**) Relative expression of mitochondrial respiratory complexes, as determined by western blot. Data are expressed as mean ± SEM of three independent experiments. (**E**,**F**) Quantification of mitochondrial membrane potential by tetramethyl-rhodamine, methyl ester (TMRM) and of anion superoxide by MTX. The net median fluorescence intensity (MFI) value of a wt sample was set to 100 and other samples were determined as relative MFI levels according to the formula: (het sample net MFI/wt sample net MFI) × 100. n = 3. Data are expressed as mean ± SEM. * = p < 0.05..

Next, we asked whether these alterations of mitochondria morphology could affect mitochondrial functionality, by evaluating OCR and ECAR of wt and het cells. We first analyzed the energetic competence of het MEFs by comparing their oxygen consumption rate and extracellular acidification rate with those of wt cells. Cells were first analyzed when cultured in a glucose containing medium, and then after culturing in glucose-free, galactose containing medium, which forces cells to use respiration rather than glycolysis. Figure 7A shows OCR and ECAR curves. MEFs from *Lonp1^wt/−^* mice cultured in glucose showed a reduced oxygen consumption rate than wt cells in any condition tested. Basal and non-mitochondrial oxygen consumption were significantly lower in het cells, while maximal respiration, despite lower than controls, did not reach statistical significance (Figure 7B). In galactose medium, both wt and het cells displayed a significant increase (around 20%) in basal respiration if compared to cells grown in glucose, and wt cells also showed a higher basal and non-mitochondrial oxygen consumption in this condition. Maximal respiration was almost identical in cells grown in glucose and galactose medium, either in the case of wt or het cells (Figure 7B). A similar pattern was observed in extracellular acidification rate: wt MEFs displayed a higher ECAR than het ones, either in glucose or galactose medium. Baseline OCR and ECAR were plotted as an energy map, showing that wt MEFs are characterized by a more energetic phenotype than het MEFs, which were more quiescent (Figure 7C). 

We wondered if the reduction of OCR we observed in het cells was due to an impairment of respiratory chain complexes. Thus, we measured complex expression levels in MEFs (Figure 7D). All complexes were detectable in het MEFs and displayed a similar reduction in comparison with wt cells. Such reduction proved significant in the case of CII, CIII and CV (Figure 7D). We also analyzed the expression of ACO2, which is a substrate of LONP1 and of Grp75, which has been shown to interact with LONP1 (Figure S6) and we did not observe any significant change. 

Since mitochondria from het cells displayed an aberrant morphology and a reduction in OCR, we wondered if this alteration could impact on mitochondrial membrane potential (MMP) and reactive oxygen species production. Thus, we measured MMP and mtO_2_^−^ levels by flow cytometry, using the MMP-sensitive probe TMRM, and mitoSOX Red, respectively. No variations were observed in both parameters in het cells, when compared to wild type ones (Figure 7E,F). No significant change was also observed in the expression of the superoxide dismutase SOD2 (Figure S6). Cells were also tested for mtO_2_^−^ production in the presence of a stressor such as H_2_O_2_ 2 mM for 30′. As expected, both wt and het cells displayed an increase in mtO_2_^−^ levels, but no differences were present between them (not shown).

## 4. Discussion

In this study we described the phenotypic and molecular characterization of a heterozygous mouse model for the protease LONP1. We confirmed the embryonic lethality of the total depletion of *Lonp1*, as previously described [32] and this result is in agreement with the observation that the silencing of *LONP1* resulted in cell death in vitro [8]. Accordingly, the numbers of wt and het litters reflect the expected Mendelian ratio we should observe in the presence of the embryonic lethality of homozygous animals. To date, no absence of *LONP1* has been described in living humans, indirectly confirming that *LONP1* is essential for cell functions. Mutations of this gene that partially alter its functionality cause CODAS syndrome, but the pathogenetic mechanisms that links genetic defect and phenotype has not been identified yet [36,39]. Conversely, het mice appear healthy, and we did not detect any gross phenotypic, nor histological alterations which could suggest the presence of inflammatory status or functional alterations of muscles, bones, nervous system and digestive tract. This is in agreement with previous studies performed on another *Lonp^wt/−^* mouse model [32,42] and with that observed in the parents of CODAS-affected patients, who appear healthy and without relevant phenotypic alterations, despite the mutation in one copy of *LONP1* [36,39,45]. 

When we analyzed *Lonp1* expression in wt and het mice, we observed a remarkable tissue variability. Few studies in the past have analyzed the expression of *LONP1* in different tissue, and mostly at the mRNA levels. High levels of LONP1 mRNA have been observed in the liver, brain, heart, skeletal muscle, and lower but clearly detectable levels in lung, kidney and pancreas. Our data showed that, as expected, *Lonp1* is highly expressed in energy-demanding, mitochondrial-dependent tissues like skeletal muscles and, to a minor extent, the liver, with the remarkable exception of colon, in which LONP1 level increases during aging. LONP1 expression has been reported in the past to undergo a progressive expression decline with aging; however, this is true only for some muscles, while the liver does not show any significant variation, and heart levels even increased in very old animals [46]. Thus, our data further confirm that LONP1 expression over time does not follow a general trend but is tissue specific. 

In most of the analyzed tissues, the het mouse expresses approximately the same amount of LONP1 protein as wt, suggesting the existence of a compensatory mechanism in the intact allele. Preliminary observations in liver, skeletal muscle and colon indicate that mRNA levels of LONP1 are increased in het mice (not shown), suggesting that this phenomenon could indeed occur. *LONP1* has been shown to be upregulated by stressors like heat shock or reactive oxygen species in several models [14,19,47]. It is possible that adaptations to altered oxidative processes in mitochondria, present when just a copy of *Lonp1* gene is functional, leads to the upregulation of the gene that (partially) compensates for the absence of one copy of the gene. Since we did not observe a higher level of mtO_2_^−^ in het MEFs, it is possible that other *Lonp1*-upregulating stimuli, such as proteotoxic stress, could determine a partial increase in *Lonp1* expression. The recent finding that the mt proteome of *Lonp1^wt/−^* MEFs obtained in another *Lonp1^wt/−^* mouse model is characterized by upregulations of pathways involved in oxidative stress and glutathione [43] is in agreement with our observation. The only two exceptions are represented by the colon and heart of adult mice, which show a significant reduction of LONP1 protein amount of approximately 50%. This is consistent with the observation of Venkatesh et al. that described a reduction of 50–60% of LONP1 level in the heart [42].

As LONP1 downregulation is associated with altered functionality and morphology of colon cancer cells in vitro and with lower risk of developing colon cancer in mice, we decided to analyze possible differences present in the colon. In particular, mitochondria of enterocytes appeared increased in number, swollen and with altered shape. Such dramatic morphological alterations are likely a direct consequence of a reduced capability of mitochondria of *Lonp1^wt/−^* cells to maintain protein homeostasis in the organelle. Indeed, our previous studies in cellular models of colorectal origin demonstrated that LONP1 silencing or pharmacological inhibition determine a severe impairment of mitochondrial proteome, leading to modification of proteins involved in stress response, energetic metabolism and respiratory chain assembly with crucial effects on mitochondrial morphology and functionality [8,48]. It is interesting to note that these effects are tissue and cell specific, as in cellular models of different origin it is greatly attenuated, and can be observed only after 10 days of LONP1 silencing [49]. Thus, it is not surprising that mitochondria of *Lonp^wt/−^* MEFs are increased in number and present morphological alterations, but in a less dramatic way than those of *Lonp^wt/−^* enterocytes. 

In MEFs, we have also found a reduction of LONP1 and of TFAM levels, in agreement with the fact that LONP1 modulates its amount, and a reduction in the amount of mtDNA and nucleoids. In their work, Quiros et al. reported that a decrease in mtDNA copy number could be observed only in LONP1-deficient embryos, while heterozygous embryos did not show significant differences from controls [32]. This discrepancy is likely due to the fact that the reduction of the mtDNA content needs a rather long period of time to take place. Indeed, no reduction of mtDNA levels can be observed in in vitro models after downregulating LONP1 to 20% of normal levels for more than 72 h [8]. Our data strengthen the idea that even if LONP1 is essential for mtDNA maintenance, a small amount of the protein is sufficient to maintain, at least partially, mtDNA copy levels in the cell for long periods.

From the functional point of view, MEFs from *Lonp^wt/−^* animals are characterized by reduced respiration capacity. Such reduction is likely mediated by a reduced presence of respiratory complexes and is in perfect agreement with what has been previously observed in different cellular models, in which *LONP1* silencing determined a dramatic reduction in respiratory capacity, accompanied by an impairment in respiratory complex formation [8,30,49]. Recent, still unpublished data obtained in a conditional knock out model of Lonp1 mouse showed decreased activity of the CI, CIII, CIV in heart tissue, further confirming that LONP1 is necessary to maintain respiratory complex functionality. However, the description of a patient with a biallelic mutation of *LONP1* with a reduced pyruvate dehydrogenase (PDH) activity and elevated intracellular lactate: pyruvate ratio suggests that the alteration of cellular metabolism in LONP1 deficient cells could also be due to mechanisms not directly related to OXPHOS impairment [41]. Since in MEFs from het mice we observed not only an impairment in respiration, but also a lower acidification rate than wt cells, we can speculate that a general decreased metabolism characterizes cells MEFs from het mice, and not a simple OXPHOS reduction. This metabolic re-modelling might be the basis of the lack of proliferative capacity of cells with a reduced expression of Lonp1 when a tumor develops.

The decrease of basal respiration could be a consequence of the decrease of non-mitochondrial respiration but could be also correlated to a minor request of ATP, consequence of the quiescent energetic phenotype showed by het MEFs. In our model, the absence of differences observed in maximal respiration could be correlated with a different method used by cells to answer the deficiency of glucose. The cause has not been examined in this study, but it has been shown that different cell lines have different adaption to galactose medium, including intensification of the synthesis of Electron transport chain (ETC) complexes, and in increase in mitochondrial biogenesis, in the use of glutamine and in processing enzyme, or a combination of each [50].

In conclusion, data from this study, and from the morphological and functional alterations observed in cells from patients with mutated *LONP1,* indicate that a reduced function of LONP1 is sufficient to impair mitochondrial ultrastructure and functions in vivo; this reduction can be the consequence either of a lower expression of the wild type protein, or of a normal expression of a partially functioning protein. In the presence of a mutation that partially reduces function, the effects of mitochondria become evident only when both alleles are mutated, while the mutation of just one allele, such as in parents of CODAS patients, is not sufficient to determine an effect. 

We are well aware that our model does not allow us to identify which, among multiple functions of LONP1, is the one that determines mitochondrial alterations when partially impaired. Further studies with a Lonp1 mouse model bearing a point mutation ablating proteolytic activity are ongoing to better clarify this point. 

## Figures and Tables

**Table 1:** Distribution of offspring in different type of mating according to sex. ^−^ indicates targeted Lonp1 allele.

		Male		Female	
Type of Mating	*Total*	Born	Expected	Born	Expected
*wt/− ♂ x wt/− ♀*	*110.0*	58.0	55.0	52.0	55.0
*wt/− ♂ x wt/wt ♀*	*67.0*	36.0	33.5	31.0	33.5
*wt/wt ♂ x wt/− ♀*	*52.0*	27.0	26.0	25.0	26.0
*Total*	*229.0*	*121.0*	*114.5*	*108.0*	*114.5*

**Table 2:** Distribution of offspring in different type of mating according to Lonp1 genotype. ^−^ indicates targeted Lonp1 allele.

		Total				Male				Female			
		Wild Type		Heterozygous		Wild Type		Heterozygous		Wild Type		Heterozygous	
Type of mating	*Total*	Born	Expected	Born	Expected	Born	Expected	Born	Expected	Born	Expected	Born	Expected
*wt/− ♂ x wt/− ♀*	*110.0*	45.0	36.7	65.0	73.3	24.0	19.3	34.0	38.7	21.0	17.3	31.0	34.7
*wt/− ♂ x wt/wt ♀*	*67.0*	33.0	33.5	34.0	33.5	21.0	18.0	15.0	18.0	12.0	15.5	19.0	15.5
*wt/wt ♂ x wt/− ♀*	*52.0*	28.0	26.0	24.0	26.0	16.0	13.5	11.0	13.5	12.0	12.5	13.0	12.5
*Total*	*229.0*	*106.0*	*96.2*	*123.0*	*132.8*	*61.0*	*50.8*	*60.0*	*70.2*	*45.0*	*45.3*	*63.0*	*62.7*

## Supplementary Materials

The following are available online at https://www.mdpi.com/2077-0383/9/6/1783/s1, Figure S1: Representation of wild-type and mutant Lonp1 alleles, and the targeting vector. Grey boxes indicate exons and lines introns- Figure S2: Comparison of the expression of LONP1 protein expression in different tissues at 2 and 8 months of age. Figure S3: Hematoxylin and eosin staining of the colon of wt and heterozygous mice. Figure S4: Cell growth curves of wt (circle) and het (square) MEFs. Figure S5: TFAM expression in MEFs as revealed by western blot. Figure S6: Analysis of the relative protein expression of ACO2, SOD2 and GRP75 in MEFs from wt and het mice as revealed by western blot.
