# Supplementary material for: Impaired Mitochondrial Morphology and Functionality in Lonp1wt/− Mice
Source: J Clin Med. 2020 Jun 8;9(6):1783. doi: 10.3390/jcm9061783 (PMC7355737; doi:10.3390/jcm9061783)
Supplement: Supplementary file 1 [file jcm-09-01783-s001.pdf]

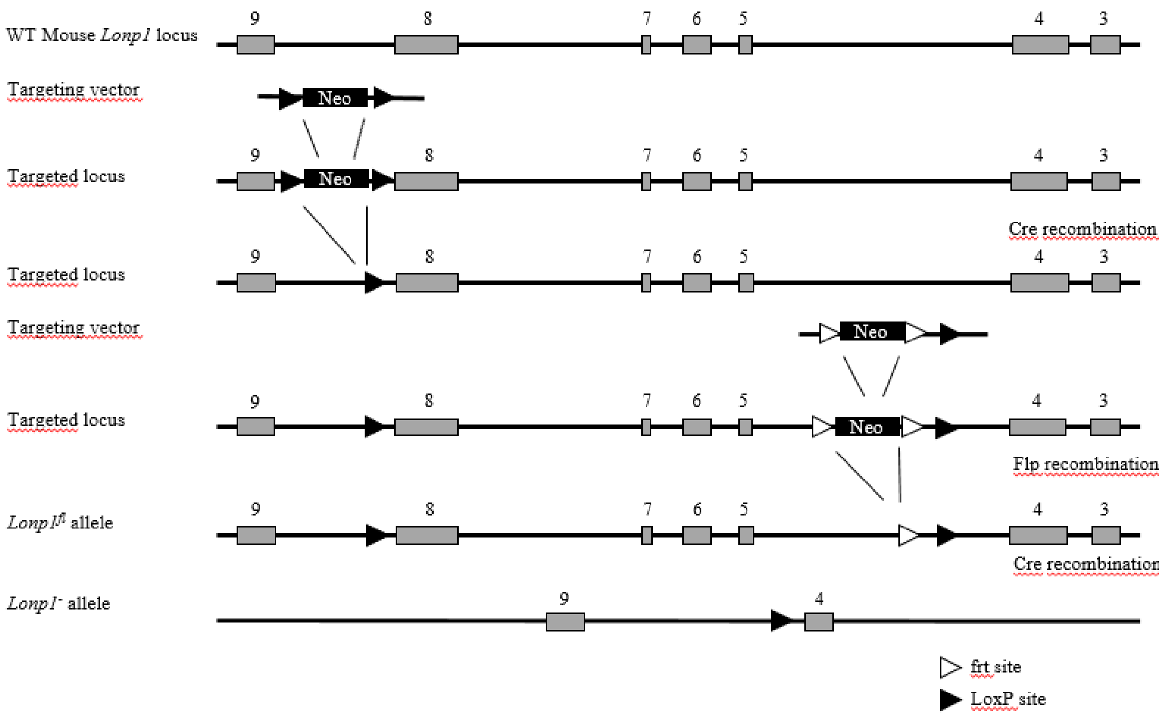

Figure 1. Representation of wild-type and mutant Lonp1 alleles, and the targeting vector. Grey boxes indicate exons and lines introns.

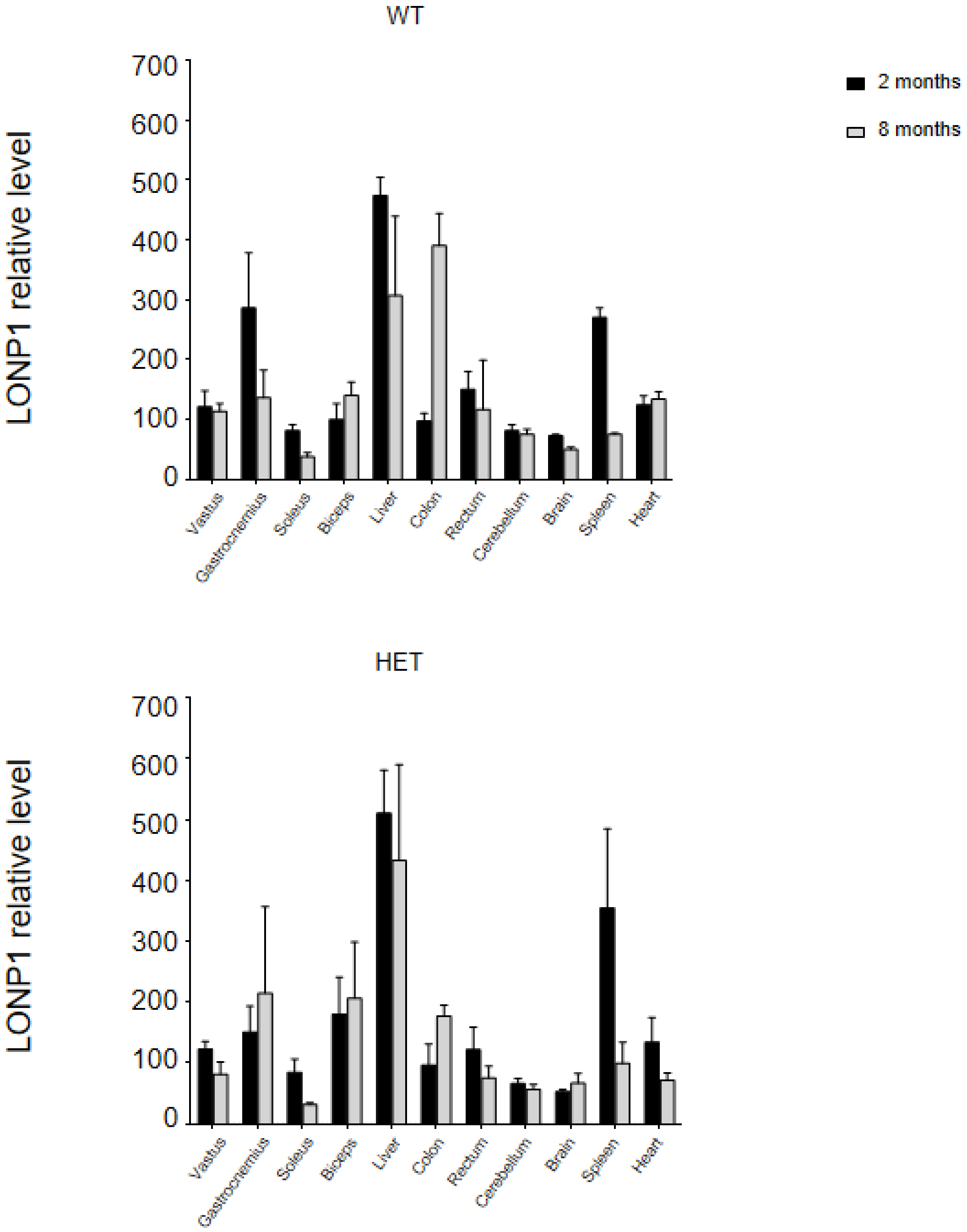

Figure 2. Comparison of the expression of LONP1 protein expression in different tissues at 2 and 8 months of age.

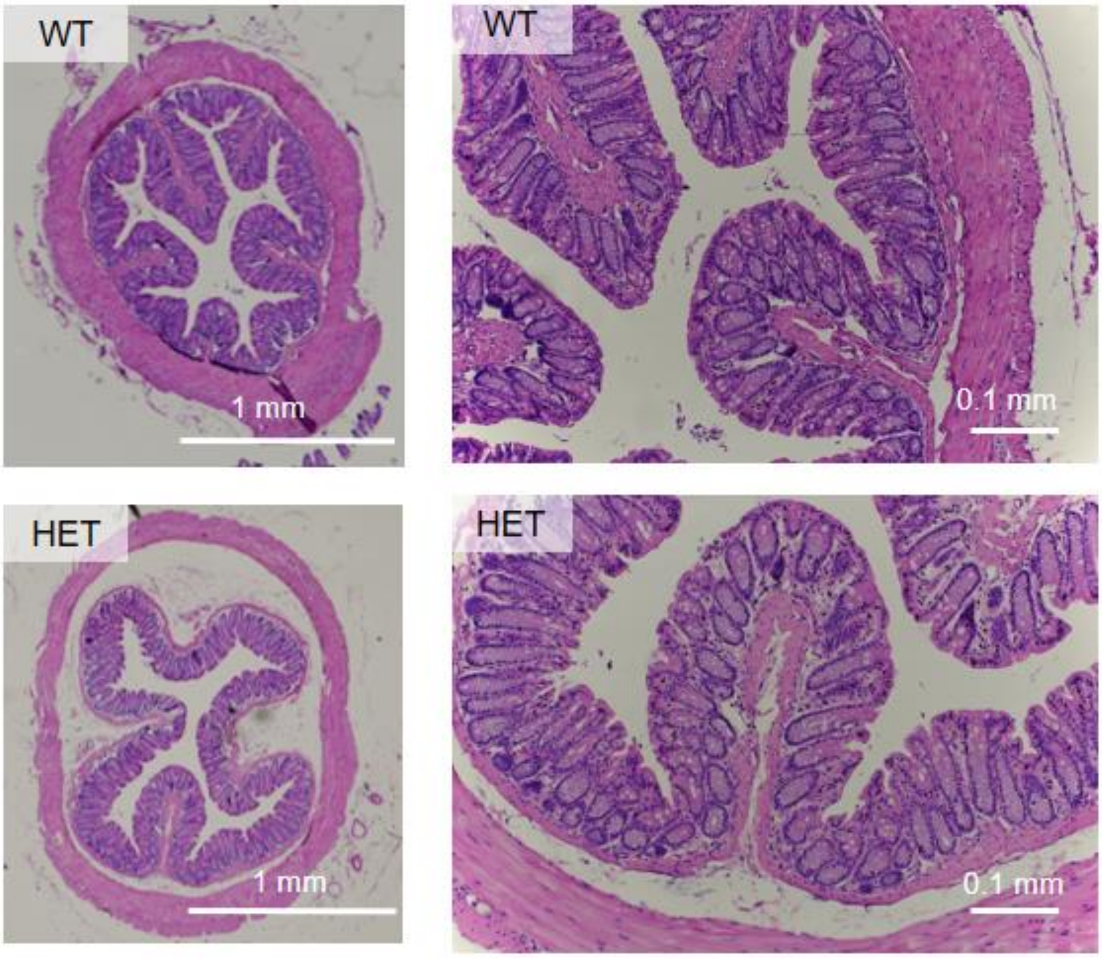

Figure 3. Hematoxylin and eosin staining of the colon of wt and heterozygous mice.

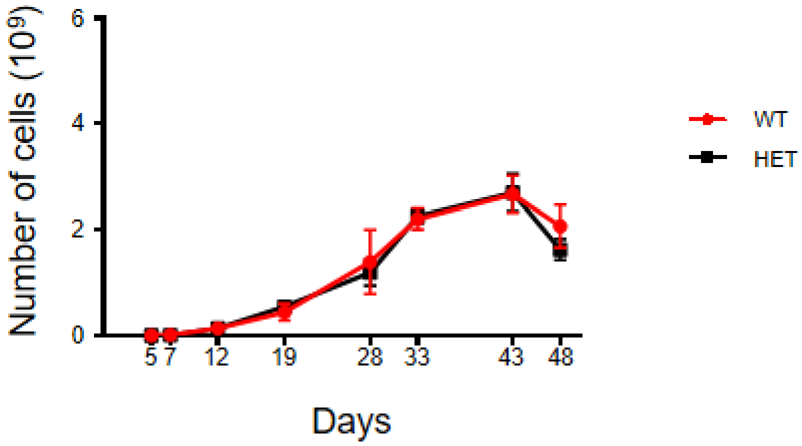

Figure 4. Cell growth curves of wt (circle) and het (square) MEFs.

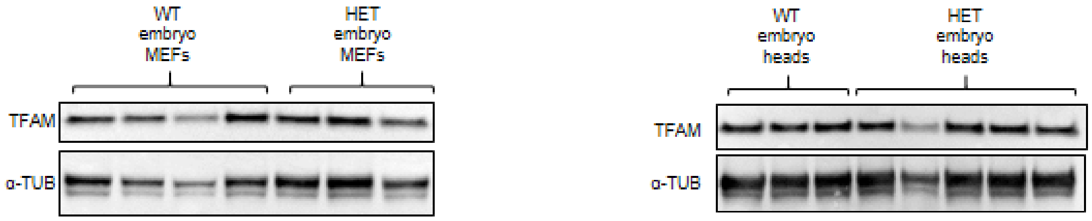

Figure 5. TFAM expression in MEFs as revealed by western blot.

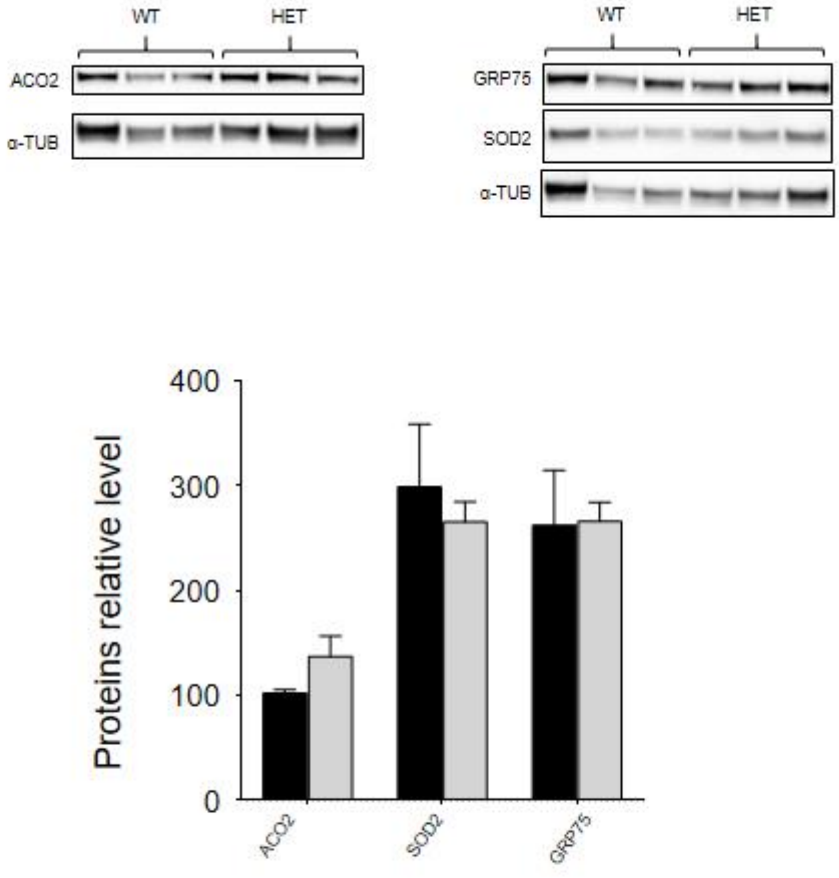

Figure 6. Analysis of the relative protein expression of ACO2, SOD2 and GRP75 in MEFs from wt and het mice as revealed by western blot.
